# The Clinical Severity of COVID-19 Variants of Concern: Retrospective Population-Based Analysis

**DOI:** 10.2196/45513

**Published:** 2024-08-27

**Authors:** Sean P Harrigan, Héctor A Velásquez García, Younathan Abdia, James Wilton, Natalie Prystajecky, John Tyson, Chris Fjell, Linda Hoang, Jeffrey C Kwong, Sharmistha Mishra, Linwei Wang, Beate Sander, Naveed Z Janjua, Hind Sbihi

**Affiliations:** 1 BC Centre for Disease Control Vancouver, BC Canada; 2 University of British Columbia Centre for Disease Control Vancouver, BC Canada; 3 Department of Pathology and Laboratory Medicine University of British Columbia Vancouver, BC Canada; 4 Institute for Clinical Evaluative Sciences Toronto, ON Canada; 5 Public Health Ontario Toronto, ON Canada; 6 Department of Family and Community Medicine University of Toronto Toronto, ON Canada; 7 Dalla Lana School of Public Health University of Toronto Toronto, ON Canada; 8 MAP Centre for Urban Health Solutions, Li Ka Shing Knowledge Institute, Unity Health Toronto Toronto, ON Canada; 9 Institute of Health Policy, Management and Evaluation Dalla Lana School of Public Health University of Toronto Toronto, ON Canada; 10 Institute of Medical Sciences University of Toronto Toronto, ON Canada; 11 Department of Medicine University of Toronto Toronto, ON Canada; 12 Toronto Health Economics and Technology Assessment Collaborative University Health Network Toronto, ON Canada; 13 School of Population and Public Health University of British Columbia Vancouver, BC Canada; 14 Centre for Advancing Health Outcomes St Paul's Hospital Vancouver, BC Canada

**Keywords:** COVID-19, SARS-CoV-2, severity, whole genome sequencing, WGS, social determinants of health, SDOHs, vaccination, variants of concern, VOCs, Alpha, Gamma, Delta, Omicron

## Abstract

**Background:**

SARS-CoV-2 variants of concern (VOCs) emerged and rapidly replaced the original strain worldwide. The increased transmissibility of these new variants led to increases in infections, hospitalizations, and mortality. However, there is a scarcity of retrospective investigations examining the severity of all the main VOCs in presence of key public health measures and within various social determinants of health (SDOHs).

**Objective:**

This study aims to provide a retrospective assessment of the clinical severity of COVID-19 VOCs in the context of heterogenous SDOHs and vaccination rollout.

**Methods:**

We used a population-based retrospective cohort design with data from the British Columbia COVID-19 Cohort, a linked provincial surveillance platform. To assess the relative severity (hospitalizations, intensive care unit [ICU] admissions, and deaths) of Gamma, Delta, and Omicron infections during 2021 relative to Alpha, we used inverse probability treatment weighted Cox proportional hazard modeling. We also conducted a subanalysis among unvaccinated individuals, as assessed severity differed across VOCs and SDOHs.

**Results:**

We included 91,964 individuals infected with a SARS-CoV-2 VOC (Alpha: n=20,487, 22.28%; Gamma: n=15,223, 16.55%; Delta: n=49,161, 53.46%; and Omicron: n=7093, 7.71%). Delta was associated with the most severe disease in terms of hospitalization, ICU admissions, and deaths (hospitalization: adjusted hazard ratio [aHR] 2.00, 95% CI 1.92-2.08; ICU: aHR 2.05, 95% CI 1.91-2.20; death: aHR 3.70, 95% CI 3.23-4.25 relative to Alpha), followed generally by Gamma and then Omicron and Alpha. The relative severity by VOC remained similar in the unvaccinated individual subanalysis, although the proportion of individuals infected with Delta and Omicron who were hospitalized was 2 times higher in those unvaccinated than in those fully vaccinated. Regarding SDOHs, the proportion of hospitalized individuals was higher in areas with lower income across all VOCs, whereas among Alpha and Gamma infections, 2 VOCs that cocirculated, differential distributions of hospitalizations were found among racially minoritized groups.

**Conclusions:**

Our study provides robust severity estimates for all VOCs during the COVID-19 pandemic in British Columbia, Canada. Relative to Alpha, we found Delta to be the most severe, followed by Gamma and Omicron. This study highlights the importance of targeted testing and sequencing to ensure timely detection and accurate estimation of severity in emerging variants. It further sheds light on the importance of vaccination coverage and SDOHs in the context of pandemic preparedness to support the prioritization of allocation for resource-constrained or minoritized groups.

## Introduction

### Background

Understanding the severity of SARS-CoV-2 variants of concern (VOCs) has been important to clinical decision-making and the implementation of appropriate public health measures. While the wild-type strain dominated infections worldwide throughout the first year of the pandemic, the sudden increase in disease severity by the end of 2020 prompted the World Health Organization to implement a program that classified SARS-CoV-2 into variants of interest and VOCs [1]. Different sources of transmission dynamics of COVID-19 in the environment [2] and risk factors in society have accelerated its diffusion and its mutations into VOCs and their current subvariants [3].

The dominant VOC has evolved throughout the pandemic, with each new variant characterized by specific mutations and viral properties, bringing new challenges to public health responses. The evolution of VOCs was marked by additional mutations to the spike protein (eg, N501Y and P681H), allowing the virus to bind more tightly to human host cells and create new spike proteins more efficiently, whereas other spike protein mutations (E484K) help evade antibodies [4]. Ultimately, additional mutational load resulted in increases in infectivity and severity as well as vaccine escape [5-11]. During 2021, there were 5 main VOCs—Alpha, Beta, Gamma, Delta, and Omicron—that circulated worldwide; however, their prevalence varied geographically. All VOCs were observed in the province of British Columbia (BC), Canada, in 2021. Alpha, Beta, and Gamma were the first to emerge in BC, and all demonstrated increased infectivity and severity compared to the wild-type strain [4-7,9,12]. This was followed by Delta and Omicron later in the year [13], resulting in unprecedented fatalities and strains to the health system, resulting in the reimplementation of many public health measures, such as social distancing [10,14].

Many studies have investigated the relative severity between 2 VOCs or a VOC and the wild-type strain [6,8]. Measuring the relative severity of VOCs over an extended period presents challenges, particularly temporal biases arising from changes in vaccination coverage and testing or sequencing strategies. For example, vaccine coverage in the year 2021 ranged from minimal to most of the population by the end of the year. Further, the prevalent VOC also changed over this time, with vaccination having been shown to be differentially effective across VOCs [15,16]. In addition to testing strategies and vaccination, public health and social measures (eg, masking and school closures) have also been shown to have an impact not only on the timing of introduction and predominance of VOCs but also on economic consequences of the VOC waves and pandemic control [17].

Social determinants of health (SDOHs) play an important role not only in disease acquisition but also in subsequent disease severity [18]. Geographic areas with lower income, lower educational attainment, and a higher proportion of essential workers and racially minoritized communities were all found to be associated with increased COVID-19 mortality in another Canadian province [19]. In the context of infectious diseases, societal risk factors may shape differential health outcomes not only through differences in contact patterns [19] but also via the extent of uptake of prevention interventions such as effective isolation and quarantine and ability to reduce nonhousehold contacts [20]. Furthermore, perceived discrimination and socioeconomic status were found to be associated with lasting COVID-19 symptoms after recovery, an important facet of the pandemic with the potential for increased burden on health care systems. Our investigation aimed to understand how SDOHs are distributed among those infected by SARS-CoV-2 when different VOCs spread in the communities and how these vary across severe clinical outcomes.

### Objectives

To date, few studies have compared the severity of >2 VOCs in a single analysis, and none has examined all VOCs in Canada. Furthermore, most evidence has relied on screening approaches based on quantitative polymerase chain reaction (qPCR) assays or on wave comparison [19,21], which can result in substantial exposure misclassification, whereas other studies have not been able to adjust for co-occurring key confounding variables, namely, SDOHs and preexisting comorbidities. In light of this, we aimed to provide robust estimates of relative severity of all prevalent VOCs in BC, Canada, from January 1, 2021, to December 31, 2021, in the context of vaccination rollout and heterogeneous SDOHs across various geographically distinct areas. This investigation may help inform pandemic preparedness by retrospectively assessing the main VOCs and the risk factors in society that can accelerate the diffusion of this virus and its variants and subvariants.

## Methods

### Sample and Data

**Figure 1:**
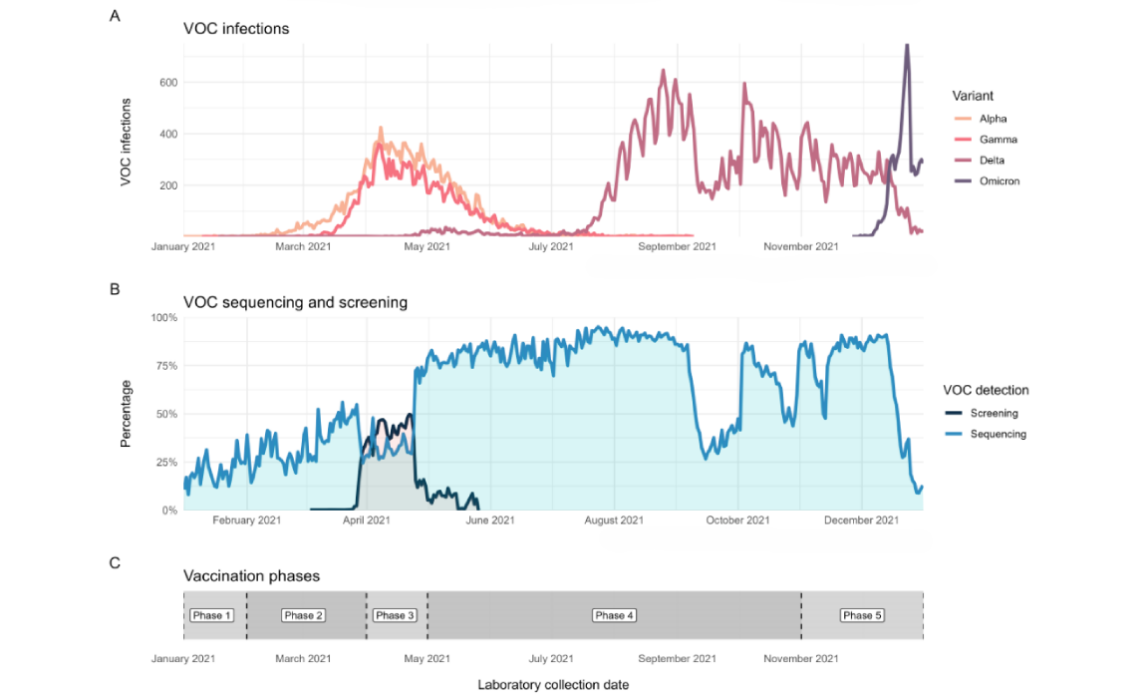
COVID-19 variant of concern (VOC) infections, sequencing, and vaccination summary for British Columbia (BC) in 2021. Vaccination phase 1 (December 2020-February 2021): eligible populations included residents, staff, and essential visitors to long-term care facilities; individuals assessed for and awaiting a long-term care placement; health care workers providing care for patients with COVID-19; and remote and isolated Indigenous communities. Vaccination phase 2 (February 2021-April 2021): eligible populations included older adults aged ≥80 years; Indigenous peoples aged ≥65 years and Indigenous Elders; Indigenous communities; hospital staff, community general practitioners, and medical specialists; vulnerable populations in select congregate settings; and staff in community home support and nursing services for older adults. Vaccination phase 3 (April 2021-May 2021): eligible populations included people aged 60 to 79 years, Indigenous peoples aged 18 to 64 years, and people aged 16 to 74 years who were clinically extremely vulnerable. Vaccination phase 4 (May 2021-November 2021): eligible populations included everyone aged ≥12 years. In September, the third dose was available for people who were clinically extremely vulnerable. Vaccination phase 5 (November 2021-February 2022): eligible populations included everyone aged ≥5 years. Children aged 5 to 11 years were eligible at the end of November. Everyone aged ≥18 years were invited to receive a booster dose within 6 to 8 months of their second dose.

During the 2021 calendar year, BC had several VOC waves as well as a vaccination program that evolved over the course of this period (Figure 1). Notably, BC experienced a unique period of cocirculation of Alpha and Gamma from March 2021 to June 2021. This was followed by a large wave of Delta infections from June 2021 to December 2021 and, finally, an even larger Omicron wave in mid-December 2021. This study period coincided with a multistage vaccination program, which ranged from only high-risk populations being vaccinated at the beginning of 2021 to nearly all adults being vaccinated at the end of 2021.

Using the BC COVID-19 Cohort (BCC19C), a population-based dynamic surveillance platform that integrates a wide range of COVID-19 data sets in addition to administrative health registries (see Table S1 in Multimedia Appendix 1 for detailed information of data holdings), our study included all BC residents diagnosed with their first COVID-19 infection [22]. Furthermore, we included only those with sequencing information (either via whole genome sequencing [WGS] or via qPCR-VOC screening) between the dates of January 1, 2021, and December 31, 2021. The end of the follow-up time was chosen based on the implementation of a new testing policy in which only individuals in hospital were tested (with the exception of international travelers), and the concomitant dissemination of rapid antigen tests for the general population, which are not captured in the surveillance system [10].

**Figure 2:**
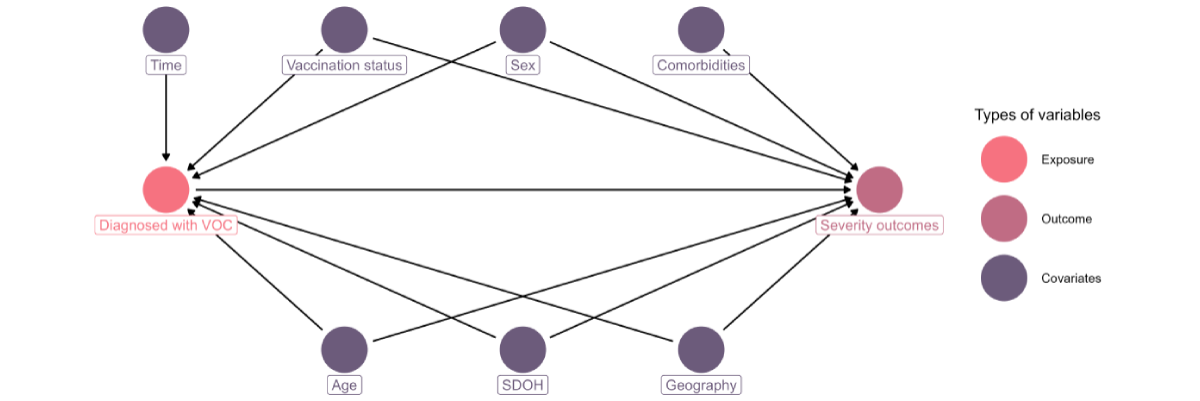
Variant of concern (VOC) severity directed acyclic graph for the British Columbia retrospective study from January 1, 2021, to December 31, 2021. VOCs are a time-dependent variable as different VOCs were present at different times throughout the study period. Health authority is a surrogate variable for differences in exposure to certain variants, public health guidelines, and health care settings. Severity outcomes include hospitalizations, intensive care unit admissions, and deaths. SDOH: social determinant of health.

We excluded all those who were infected with Beta (as this variant accounted for <0.1% of all cases during the study period), resided outside BC, were admitted to hospital at least 2 days before positive collection date, did not appear in BC’s health insurance data set (Medical Services Plan), were aged <2 years (to exclude anyone born after the start of the pandemic), were living in long-term care, and had missing data, as well as observations with other data quality issues such as missing hospital admission date and death before laboratory collection date or hospital admission date (Figure 2).

### Measure of Variables

The VOCs were identified at the BC Centre for Disease Control (BCCDC) Public Health Laboratory using mostly WGS for sample characterization but also having relied on VOC screening through qPCR [23] as the provincial laboratory optimized its sequencing strategy based on available laboratory capacity and public health needs (see Table S2 in Multimedia Appendix 1 for the sequencing and screening phases). For samples that underwent both qPCR single-nucleotide polymorphism screening and further confirmation through WGS, lineage results from WGS were used. VOC assignment for all samples in this study was performed after using standardized and validated library preparation and Illumina-based sequencing protocols [24] producing consensus genomic outputs. Only samples with ≥85% sequence coverage and no quality control flags in the bioinformatics pipeline were included [25].

In total, 3 outcomes of interest were considered: hospitalization, intensive care unit (ICU) admission, and death. *Hospitalization* was defined as an admission from 2 days before to 14 days after the positive COVID-19 sample collection. This definition has been used in other VOC severity–related studies as well as in our previous study investigating Delta and Omicron severity during a short period of cocirculation [10,13,26,27]. *ICU admission* was defined as anyone who met the criteria for hospitalization and who was also admitted to the ICU during that hospital admission. Finally, *death* was defined as those who had met the hospitalization criteria and died within 30 days of laboratory collection date. More detailed descriptions can be found in Table S3 in Multimedia Appendix 1.

Individual-level data were extracted for age, sex (male or female), and geography of residence (5 BC regional health authorities) from the BC case surveillance data set integrated into the BCC19C. If any of these variables were missing, they were supplemented from the client roster. Vaccination status was determined via the Provincial Immunization Registry, which provides a record of the dates and agent of each dose received. Vaccinations were required to be received at least 14 days before COVID-19 infection to be considered toward vaccination status. For the purposes of analysis, vaccination status was categorized as unvaccinated, partially vaccinated, and fully vaccinated (*fully vaccinated* indicating individuals who received at least one dose of Johnson & Johnson or 2 doses of any other vaccine). During 2021 in BC, the booster dose program started in late December, and only limited numbers of high-risk individuals had received boosters; therefore, booster doses were not considered separately for analysis.

The Elixhauser Comorbidity Index [28] was used to measure comorbidities. This index, which provides a cumulative score of 1, 2, or ≥3 comorbidities, is calculated based on hospitalization discharge data and physician billing before an individual’s positive COVID-19 laboratory collection date. Finally, SDOH data were extracted at the dissemination area level, the smallest unit of geographic area (representing 400-700 residents) used in Canadian census data, from the 2016 census and was linked to each individual by residential postal code. We considered the following SDOH variables: neighborhood income, proportion of essential workers, proportion of racially minoritized groups, and proportion of high-density housing [19,29]. Each SDOH variable was converted to quintiles, with quintile 5 being the most deprived (see Table S3 in Multimedia Appendix 1 for more details and variable definitions).

### Data Analysis Procedure

Descriptive analyses were initially conducted to understand distributions of key confounding variables across VOCs. Figure 2 shows the directed acyclic graph that informed study design and statistical analysis. To examine the relative severity of VOCs, Cox proportional hazard models weighted with inverse probability treatment weights (IPTWs) were used to assess the severity of VOCs on each of the 3 severe outcomes.

IPTWs weigh individuals on the inverse of the probability of being exposed to a VOC given measured confounders using a multinomial logistic regression model. These weights were then used in weighted Cox regression models adjusting for the confounders used in the weight creation. For the IPTW weight calculation, we included the following variables: age, sex, health authority, comorbidities, income, proportion of essential workers, proportion of racially minoritized groups, and proportion of high-density housing. Vaccination was not included in the weight creation due to the time-varying nature of both vaccination and VOCs and, therefore, was only adjusted for in the final weighted regression. To assess covariate balance after weighting, a stringent standardized mean difference threshold of 0.10 was adopted to determine imbalance [30-33]. We further conducted a subanalysis for severity limited to only unvaccinated individuals. For outcome counts of <10, we did not present model estimates or counts as we were required to mask results for identification purposes. Finally, to understand the potential interactions between VOCs and SDOH measures in COVID-19 severity outcomes, we examined the distribution of each SDOH among infected and hospitalized individuals and their ratio overall and within each VOC stratum.

Sensitivity analyses were also conducted to account for incidental hospitalizations. We narrowed the outcome definition from −2 to 14 days from laboratory collection date to 0 to 14 days and 1 to 14 days to exclude infections that may have been acquired in hospital. All individuals who did not meet this new hospitalization definition were excluded from the sensitivity analysis.

### Ethical Considerations

This study was reviewed and approved by the Behavioural Research Ethics Board at the University of BC (H20-02097). Analyses of severe outcomes were undertaken under the BCCDC population health surveillance and risk assessment mandate. Analyses used health care administrative data collected as part of routine care, which does not require participant consent. Deidentified data are available through the BCC19C for analyses. The BCC19C was established and is maintained through operational support from Data Analytics, Reporting, and Evaluation and the BCCDC at the Provincial Health Services Authority [22].

## Results

### Study Population: Inclusions and Exclusions

**Figure 3:**
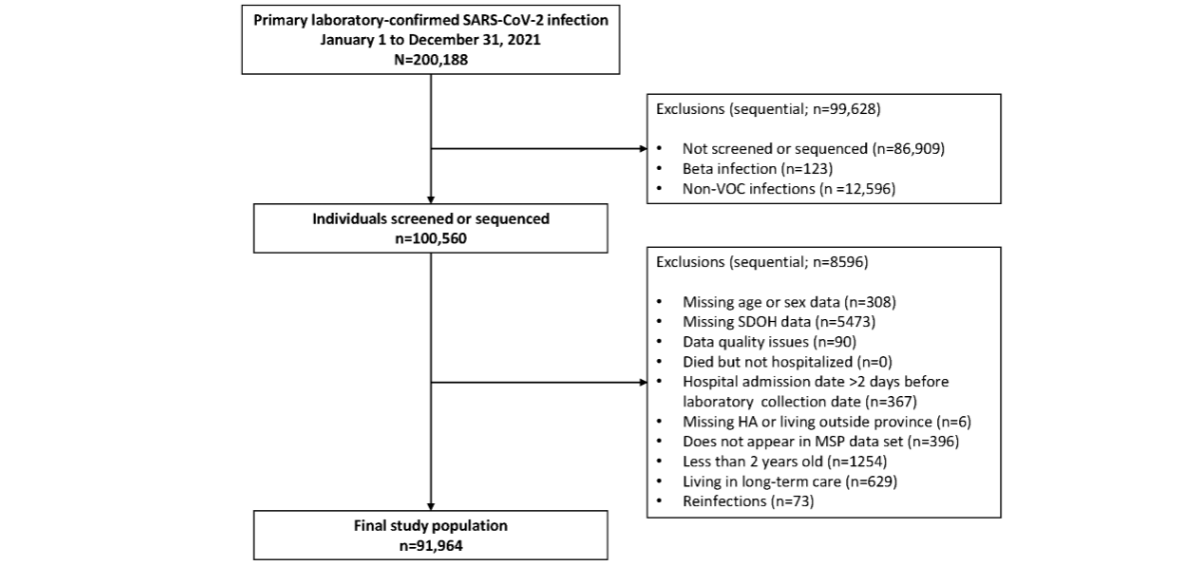
Study flow diagram of the British Columbia (BC) analytical cohort examined between January 1, 2021, and December 31, 2021. Data quality issues included missing hospital admission date and death before laboratory collection date or hospital admission date. HA: health authority; MSP: Medical Services Plan (BC’s universal health insurance); SDOH: social determinant of health; VOC: variant of concern.

Overall, 200,188 individuals were diagnosed with their first laboratory-confirmed SARS-CoV-2 infection between January 1, 2021, and December 31, 2021. We excluded 43.41% (86,909/200,188) of people whose diagnostic specimen was not sequenced or screened during this period (as per the sequencing strategy, this represented an unbiased sample of the total population of infected individuals) and 0.06% (123/200,188) of individuals who were infected with the Beta variant (due to small sample size). A further 4.29% (8596/200,188) of individuals were excluded due to data quality issues. After all exclusions, there were 91,964 individuals included in the study (Figure 3).

### Population Demographics

From the 91,964 individuals with characterized VOC infection, the cohort included 20,487 (22.28%) individuals infected with Alpha, 15,223 (16.55%) infected with Gamma, 49,161 (53.46%) infected with Delta, and 7093 (7.71%) infected with Omicron (Table 1). VOC was confirmed through WGS in 86.6% (79,640/91,964) of individuals, and the remaining 13.4% (12,324/91,964) of infections were determined through qPCR single-nucleotide polymorphism screening. The average age was 35.9 (SD 18.9; median 34.0) years. Age distributions varied by VOC, with individuals infected with Omicron generally being younger in age (see Table S4 in Multimedia Appendix 1 for age group distribution). There was roughly an equal proportion of male and female individuals (45,275/91,964, 49.23% were female) overall, and this remained similar by VOC. Over 60% of BC residents infected with a VOC lived in the Lower Mainland of BC, the largest population center in the province. Table S4 in Multimedia Appendix 1 shows the total population in the Lower Mainland, BC, which comprises the Vancouver coastal and Fraser regions (58,536/91,964, 63.65%). Over one-third of individuals (34,355/91,964, 37.36%) had no comorbidities, whereas one-fifth (18,667/91,964, 20.29%) had ≥3.

Vaccination status also differed across VOCs, largely due to the time-varying nature in circulating VOCs and vaccination rollout. For individuals infected with Alpha and Gamma, only 0.37% (75/20,487) and 0.46% (70/15,223) received their full primary series doses, respectively, whereas 29.61% (14,558/49,161) of Delta infections and 91.01% (6456/7093) of Omicron infections were among fully vaccinated individuals.

**Table 1:** Study population summary demographics from January 1, 2021, to December 31, 2021, in British Columbia, Canada (N=91,964).

Demographics		Alpha (n=20,487)	Gamma (n=15,223)	Delta (n=49,161)	Omicron (n=7093)	Overall (N=91,964)
**Sex, n (%)**						
	Female	10,075 (49.18)	7208 (47.34)	24,386 (49.6)	3606 (50.84)	45,275 (49.23)
	Male	10,412 (50.82)	8015 (52.66)	24,775 (50.4)	3487 (49.16)	46,689 (50.77)
**Age (y)**						
	Mean (SD)	36.6 (18.7)	36.1 (18.0)	35.7 (19.6)	35.5 (16.0)	35.9 (18.9)
	Median (range)	35.0 (3.0-107)	34.0 (3.0-102)	34.0 (3.0-104)	33.0 (3.0-100)	34.0 (3.0-107)
**Vaccination status, n (%)**						
	Not vaccinated	18,809 (91.81)	13,612 (89.42)	30,448 (61.94)	526 (7.43)	63,395 (68.93)
	Partially vaccinated	1603 (7.82)	1541 (10.12)	4155 (8.55)	111 (1.56)	7410 (8.06)
	Fully vaccinated	75 (0.37)	70 (0.46)	14,558 (29.61)	6456 (91.01)	21,159 (23.01)
**Comorbidities, n (%)**						
	0	7624 (37.21)	6151 (40.41)	17,928 (36.47)	2652 (37.39)	34,355 (37.36)
	1	5494 (26.82)	4180 (27.46)	12,990 (26.42)	2141 (30.19)	24,805 (26.97)
	2	3227 (15.75)	2232 (14.66)	7523 (15.30)	1155 (16.28)	14,137 (15.37)
	≥3	4142 (20.22)	2660 (17.47)	10,720 (21.82)	1145 (16.14)	18,667 (20.3)
**Hospitalization, n (%)**						
	No hospital admission	19,499 (95.18)	14,159 (93.01)	45,971 (93.51)	6983 (98.45)	86,612 (94.18)
	Hospital admission	988 (4.82)	1064 (6.99)	3190 (6.49)	110 (1.55)	5352 (5.82)
**ICU^a^ admission, n (%)**						
	No ICU admission	20,193 (98.57)	14,873 (97.7)	48,139 (97.92)	7083 (99.86)	90,288 (98.18)
	ICU admission	294 (1.43)	350 (2.3)	1022 (2.08)	10 (0.14)	1676 (1.82)
**Death, n (%)**						
	Alive	20,416 (99.65)	15,161 (99.59)	48,734 (99.13)	7088 (99.92)	91,399 (99.39)
	Died	71 (0.35)	62 (0.41)	427 (0.87)	<10^b^	565 (0.61)
**Income, n (%)**						
	Quintile 1 (highest)	3081 (15.04)	2958 (19.43)	9164 (18.64)	1761 (24.83)	16,964 (18.45)
	Quintile 5	4151 (20.26)	3024 (19.87)	10,828 (22.03)	1010 (14.24)	19,013 (20.67)
**Essential worker proportion, n (%)**						
	Quintile 1 (lowest)	2724 (13.3)	3225 (21.19)	6061 (12.33)	2071 (29.2)	14,081 (15.31)
	Quintile 5	6265 (30.58)	2802 (18.41)	11,626 (23.62)	774 (10.91)	21,467 (23.34)
**Racially minoritized group proportion, n (%)**						
	Quintile 1 (lowest)	1038 (5.07)	662 (4.35)	10,997 (22.37)	537 (7.57)	13,234 (14.39)
	Quintile 5	9600 (46.86)	5504 (36.16)	6378 (12.97)	1904 (26.84)	23,386 (25.43)
**High-density housing proportion, n (%)**						
	Quintile 1 (lowest)	1613 (7.87)	1257 (8.26)	9505 (19.33)	796 (11.22)	13,171 (14.32)
	Quintile 5	6354 (31.02)	5171 (33.97)	8925 (18.15)	2087 (29.42)	22,537 (24.51)

^a^ICU: intensive care unit.

^b^For values <10, we did not present model estimates or counts.

### Descriptive Results by Severe Outcomes and by SDOH

Overall, there were 5352 COVID-19 hospitalizations, with 988 (18.46%) patients infected with Alpha (proportion of hospitalized individuals: 988/20,487, 4.82%), 1065 (19.9%) infected with Gamma (proportion of hospitalized individuals: 1065/15,223, 7%), 3190 (59.6%) infected with Delta (proportion of hospitalized individuals: 3190/49,161, 6.49%), and 110 (2.06%) infected with Omicron (proportion of hospitalized individuals: 110/7093, 1.55%). There were also 1676 ICU admissions, with the highest proportion of individuals admitted to the ICU being infected with Gamma and Delta (350/15,223, 2.3% and 1022/49,161, 2.08%, respectively), whereas the lowest proportion was infected with Omicron (10/7093, 0.14%). Finally, 565 deaths occurred in the cohort, with the largest proportion having died of Delta infections (427/49,161, 0.87%), twice that of Gamma (62/15,223, 0.41%).

Distributions of the number of infections varied considerably by SDOH (see Table S4 in Multimedia Appendix 1 for all quintile distributions for each SDOH), with the largest number of infections coming from areas in the lowest income quintile (19,013/91,964, 20.67%), with the highest proportion of essential workers (21,467/91,964, 23.34%), with the highest proportion of racially minoritized groups (23,386/91,964, 25.43%), and with the highest proportion of high-density housing (22,537/91,964, 24.51%). SDOH heterogeneity was also observed within VOC strata, with opposing distribution trends being observed in Omicron and Alpha for income and for proportion of essential workers. For the cocirculating Alpha and Gamma VOCs, almost 50% of Alpha infections (9600/20,487, 46.86%) were in the quintile with the highest proportion of racially minoritized groups (quintile 5).

When further examining SDOHs among those hospitalized, marked differences were found by income, proportion of essential workers, proportion of racially minoritized group, and housing density (Table 2). Specifically, the proportion of hospitalized individuals increased with decreasing income (quintile 1: 713/16,964, 4.2%; quintile 5: 1597/19,013, 8.4%), increasing proportion of essential workers (quintile 1: 665/14,081, 4.72%; quintile 5: 1422/21,467, 6.62%), and increasing proportion of high-density housing (quintile 1: 717/13,171, 5.44%; quintile 5: 1455/22,537, 6.46%). The opposite trend was observed for the proportion of racially minoritized groups, with a decreasing proportion of hospitalized individuals when moving from lowest to highest quintile (quintile 1: 904/13,234, 6.83%; quintile 5: 1306/23,386, 5.58%). When stratifying by VOC, the same trends held for income, proportion of essential workers, and proportion of high-density housing. For Alpha, Gamma, and Delta, there was approximately a 2-fold increase in the proportion hospitalized from the lowest to the highest income quintile, whereas it was almost 3-fold for Omicron.

**Table 2:** Infections and proportion of hospitalized individuals by variant of concern (VOC) and social determinant of health (SDOH) between January 1, 2021, and December 31, 2021, in British Columbia, Canada.

SDOH quintile^a^		SDOH variable			
		Income (quintile 1=highest; hospitalizations/infections)	Essential workers (quintile 1=lowest; hospitalizations/infections)	Racially minoritized groups (quintile 1=lowest; hospitalizations/infections)	High-density housing (quintile 1=lowest; hospitalizations/infections)
**Overall (5352/91,964, 5.82%)**					
	Quintile 1	713/16,964 (4.2)	665/14,081 (4.72)	904/13,234 (6.83)	717/13,171 (5.43)
	Quintile 2	879/18,771 (4.68)	934/17,894 (5.22)	1075/17,523 (6.14)	835/16,231 (5.14)
	Quintile 3	1003/18,541 (5.41)	1017/19,058 (5.34)	1021/18,189 (5.61)	1125/19,101 (5.89)
	Quintile 4	1160/18,675 (6.21)	1314/19,464 (6.75)	1046/19,632 (5.32)	1220/20,924 (5.83)
	Quintile 5	1597/19,013 (8.4)	1422/21,467 (6.62)	1306/23,386 (5.58)	1455/22,537 (6.46)
**Stratified**					
	*P* value^b^	<.001	<.001	<.001	<.001
**Alpha (988/20,487, 4.82%)**					
	Quintile 1	113/3081 (3.67)	117/2724 (4.29)	77/1038 (7.42)	92/1613 (5.7)
	Quintile 2	151/3611 (4.18)	157/3506 (4.48)	115/1886 (6.1)	118/2590 (4.56)
	Quintile 3	215/4394 (4.89)	182/3884 (4.69)	138/3016 (4.58)	183/3910 (4.68)
	Quintile 4	257/5250 (4.89)	230/4108 (5.6)	218/4947 (4.41)	277/6020 (4.6)
	Quintile 5	252/4151 (6.07)	302/6265 (4.82)	440/9600 (4.58)	318/6354 (5.01)
**Gamma (1064/15,223, 6.99%)**					
	Quintile 1	161/2958 (5.44)	210/3225 (6.5)	40/662 (6.04)	61/1257 (4.85)
	Quintile 2	178/3174 (5.61)	247/3512 (7.03)	103/1792 (5.75)	117/2090 (5.6)
	Quintile 3	202/3094 (6.53)	219/3008 (7.28)	151/2853 (5.29)	182/2838 (6.41)
	Quintile 4	245/2973 (8.24)	178/2676 (6.65)	306/4412 (6.94)	307/3867 (7.94)
	Quintile 5	278/3024 (9.19)	210/2802 (7.5)	464/5504 (8.43)	397/5171 (7.68)
**Delta (3190/49,161, 6.49%)**					
	Quintile 1	423/9164 (4.62)	313/6061 (5.16)	780/10,997 (7.09)	553/9505 (5.82)
	Quintile 2	522/10,297 (5.07)	510/9112 (5.6)	849/12,967 (6.55)	589/10,430 (5.65)
	Quintile 3	567/9630 (5.89)	597/10,729 (5.56)	705/10,824 (6.51)	739/10,901 (6.78)
	Quintile 4	644/9242 (6.97)	880/11,633 (7.56)	494/7995 (6.18)	610/9400 (6.49)
	Quintile 5	1034/10,828 (9.55)	890/11,626 (7.66)	362/6378 (5.68)	699/8925 (7.83)
**Omicron, n=100/N=7,093 (1.41%)**					
	Quintile 1	16/1761 (0.91)	25/2071 (1.21)	7/537 (1.3)	11/796 (1.38)
	Quintile 2	28/1689 (1.66)	20/1764 (1.13)	8/878 (0.91)	11/1121 (0.98)
	Quintile 3	19/1423 (1.34)	19/1437 (1.32)	27/1496 (1.8)	21/1452 (1.45)
	Quintile 4	14/1210 (1.16)	26/1047 (2.48)	28/2278 (1.23)	26/1637 (1.59)
	Quintile 5	33/1010 (3.27)	20/774 (2.58)	40/1904 (2.1)	41/2087 (1.96)

^a^Quintile 1 is that with the highest income, lowest proportion of essential workers, lowest proportion of racially minoritized groups, and lowest proportion of high-density housing.

^b^The *P* value represents a chi-square test for the hospitalization counts among VOCs within a certain SDOH.

### Multivariable Regression Analysis of Severe Outcomes

Compared to Alpha, Delta was associated with the highest relative severity in terms of hospitalization (adjusted hazard ratio [aHR] 2.00, 95% CI 1.92-2.08), followed by Omicron (aHR 1.46, 95% CI 1.36-1.56) and Gamma (aHR 1.43, 95% CI 1.38-1.49), both having similar severity (Table 3). Results were similar for the unvaccinated subcohort, with Delta again being the most severe (Delta: aHR 2.03, 95% CI 1.94-2.12; Omicron: aHR 1.55, 95% CI 1.47-1.63; Gamma: aHR 1.44, 95% CI 1.73-1.51).

Regarding ICU admissions, again Delta was the most severe (aHR 2.05, 95% CI 1.91-2.20), followed by Gamma (aHR 1.80, 95% CI 1.69-1.93), whereas Omicron had the lowest relative severity (aHR 0.73, 95% CI 0.61-0.86). In the unvaccinated subcohort, the relative severity of Delta was slightly more pronounced (Delta: aHR 2.09, 95% CI 1.93-2.26), again followed by Gamma (aHR 1.68, 95% CI 1.55-1.82). Results for Omicron were not reported due to outcome counts of <10.

**Table 3:** Inverse probability treatment weighted Cox proportional hazard models for severity outcomes from January 1, 2021, to December 31, 2021, in British Columbia, Canada.

Stratification and variant				Hospitalized/infected, n/N (%)		Adjusted hazard ratio^a^ (95% CI)
**Hospitalizations**						
	**Fully vaccinated**					
		Alpha	988/20,487 (4.82)		Reference	
		Gamma	1064/15,223 (6.99)		1.43 (1.38-1.49)	
		Delta	3190/49,161 (6.48)		2.00 (1.92-2.08)	
		Omicron	110/7093 (1.55)		1.46 (1.36-1.56)	
	**Unvaccinated**					
		Alpha	866/18,809 (4.6)		Reference	
		Gamma	915/13,612 (6.72)		1.44 (1.37-1.51)	
		Delta	2441/30,448 (8.02)		2.03 (1.94-2.12)	
		Omicron	15/526 (2.85)		1.55 (1.47-1.63)	
**ICU^b^ admissions**						
	**Fully vaccinated**					
		Alpha	294/20,487 (1.43)		Reference	
		Gamma	350/15,223 (2.3)		1.80 (1.69-1.93)	
		Delta	1022/49,161 (2.08)		2.05 (1.91-2.20)	
		Omicron	10/7093 (0.14)		0.73 (0.61-0.86)	
	**Unvaccinated**					
		Alpha	267/18,809 (1.4)		Reference	
		Gamma	323/13,612 (2)		1.68 (1.55-1.82)	
		Delta	864/30,448 (2.8)		2.09 (1.93-2.26)	
		Omicron	*<10/526* (*masked*)^c^		Masked	
**Deaths**						
	**Fully vaccinated**					
		Alpha	71/20,487 (0.35)		Reference	
		Gamma	62/15,223 (0.41)		1.33 (1.15-1.54)	
		Delta	427/49,161 (0.87)		3.70 (3.23-4.25)	
		Omicron	*<10/7093* (*masked*)		Masked	
	**Unvaccinated**					
		Alpha	49/18,809 (0.26)		Reference	
		Gamma	43/13,612 (0.32)		1.42 (1.16-1.73)	
		Delta	295/30,448 (0.97)		3.51 (2.94-4.20)	
		Omicron	*<10/526* (*masked*)		Masked	

^a^All variables in both the fully vaccinated and unvaccinated subcohorts had standardized mean differences below the threshold.

^b^ICU: intensive care unit.

^c^All results that were required by the data stewards (Ministry of Health) to be masked for privacy concerns are italicized.

Finally, regarding deaths, Delta was again the most severe (aHR 3.70, 95% CI 3.23-4.25) with more than a 3-fold increase in risk of death, followed by Gamma (aHR 1.33, 95% CI 1.15-1.54). In the unvaccinated subcohort, Delta was again the most severe (aHR 3.51, 95% CI 2.94-4.20), again followed by Gamma (aHR 1.42, 95% CI 1.16-1.73). Similar to ICU admission, Omicron results were not reported due to small outcome counts. Full models for hospitalizations, ICU admissions, and deaths are provided for the full cohort in Table S5 in Multimedia Appendix 1.

### Sensitivity Analysis

Our sensitivity analysis of the hospitalization definition showed that excluding hospitalizations that occurred 1 to 2 days before laboratory collection date (128/5352, 2.39% of hospitalizations) resulted in very similar effect estimates (Table S6 in Multimedia Appendix 1). Further excluding those hospitalized on the same day as laboratory collection date (1738/5352, 32.47%) resulted in the total exclusion of 2190 events. This resulted in an increase in the relative severity of Gamma and a decrease in severity for Delta infections. However, it is important to note that, with this strict criterion, we excluded >40% (2190/5352, 40.92%) of hospitalizations.

## Discussion

### Principal Findings

In this retrospective analysis of 91,964 individuals diagnosed with COVID-19, VOCs were characterized between January 1, 2021, and December 31, 2021, with the following distribution: 20,487 (22.28%) individuals were infected with Alpha, 15,223 (16.55%) were infected with Gamma, 49,161 (53.46%) were infected with Delta, and 7093 (7.71%) were infected with Omicron.

Differential distributions of infections and hospitalizations were observed across SDOHs. Overall, areas with a lower neighborhood-level income, higher proportion of essential workers, and higher proportion of high-density housing generally had higher number of infections and hospitalizations, reflecting that those living in more marginalized areas were inequitably affected by the COVID-19 pandemic [19]. However, differences were observed for specific VOCs. Notably, neighborhood areas with a higher proportion of visible minority groups had less hospitalizations when the Delta VOC was circulating compared to neighborhood with less visible minority groups, which was opposite to all other VOCs.

In light of such differences observed in the distribution of SDOHs by VOC, we used IPTW analysis to rigorously adjust for age, sex, comorbidities, geography, and vaccination status, as well as for highly granular neighborhood-level SDOH measures, including income, proportion of essential workers, proportion of high-density housing, and racially minoritized group profiles. Given that SDOHs and severe COVID-19 outcomes both affect likelihoods of being diagnosed or hospitalized, our IPTW approach and further adjustment for covariates would reduce biases in the estimates of risks of hospitalization, ICU admission, and death. Inclusion of several SDOHs, which are typically examined in silos, may provide further insights on how SARS-CoV-2 VOCs affect population subgroups differentially and lead to severe outcomes.

In the overall cohort, Delta was generally found to be the most severe VOC, especially in terms of ICU admissions and deaths. Gamma and Omicron were found to have similar relative severities in terms of hospitalizations, both being more severe than Alpha; however, regarding ICU admissions, Omicron had the lowest relative severity. For Omicron infections, we had few (<10) deaths recorded and were, therefore, required to mask our results for this VOC; however, one large British study found Omicron to be associated with a 3-fold decrease in risk of death compared to Delta (aHR 0.31, 95% CI 0.26-0.37) [12]. Our study shed some light on the differences in severe outcomes between the overall cohort and the unvaccinated subcohort as we demonstrated that the proportion of hospitalized individuals with Delta or Omicron (VOCs that circulated when vaccination was prevalent) was higher among this unvaccinated subcohort, exemplifying the importance of continued vaccination. Importance of vaccination worldwide has been noted in several studies [34-36] in reducing COVID-19 severity and mortality, notwithstanding the importance of combining these efforts with nonpharmaceutical interventions [37,38].

Our results align with those of other severity studies, although only a few have directly compared all 4 VOCs in the same analysis. Most other studies have compared 2 VOCs, generally with a non-VOC as the reference [9,39-41]. In a meta-analysis by Lin et al [5], researchers compared 26 studies to assess the relative severity of Alpha, Beta, Gamma, and Delta against the wild-type strain. The analysis found Delta to be the most severe, followed by Gamma and then Alpha in terms of hospitalizations and ICU admissions when compared to the wild-type strain (hospitalization aHR 2.08, 95% CI 1.77-2.39; ICU admission aHR 3.35, 95% CI 2.5-4.2). They also found Delta to be the most severe in terms of deaths (aHR 2.33, 95% CI 1.45-3.21), results that mirrored those of our analysis. Although this meta-analysis used non-VOC strains as the reference group, it does allow us to make relative comparisons between VOCs. Comparisons to this meta-analysis may be challenged by differences in outcome definitions, exposure measurement (WGS vs screening vs temporal classification), reference group (non-VOC vs Alpha), or covariates adjusted for in the models (comorbidities and SDOHs). However, this meta-analysis was published before the emergence of Omicron and, therefore, was not able to identify its relative severity. Nonetheless, our previous study found Omicron to be approximately half as severe as Delta in terms of hospitalizations [10]. Our study is further able to fill this gap in the literature relative to other prevalent VOCs.

Over the length of this study period, the vaccination landscape in BC changed dramatically—from single doses for high-risk individuals in January 2021 to most individuals being fully vaccinated by December 2021. Concurrently, exposure to specific VOCs was also heavily time dependent, supporting the decision to analyze a subcohort of unvaccinated individuals (a subanalysis of fully vaccinated individuals was not possible to due small sample sizes in Alpha and Gamma infections). For instance, the higher proportion of hospitalized individuals observed in the unvaccinated substrata with Alpha and Gamma infections was likely due to the vaccination strategy prioritizing high-risk individuals. This is contrasted by higher proportions of hospitalized individuals with Delta and Omicron, in which most individuals were fully vaccinated. Although our study was not designed to examine vaccine effectiveness, we did observe the largest vaccine breakthrough among Omicron infections (6456/7093, 91.02% of infections being among those fully vaccinated); however, it remains important to point out that, among these breakthrough infections, protection against hospitalization remained (proportion of hospitalized individuals: vaccinated 110/7093, 1.55%; unvaccinated 15/526, 2.85%). These results further shed light on the importance of vaccination and booster dose programs.

SDOHs include a wide array of factors that can influence COVID-19 infection and severity; therefore, examining the relationship between SDOHs and VOCs is paramount. We found distributions of hospitalizations to be similar across VOCs for certain SDOH variables (eg, lower-income areas with a higher proportion of hospitalized individuals), whereas hospitalization distributions varied across VOCs for other SDOHs (eg, proportion of racially minoritized groups). Our results indicate that both VOCs and SDOHs as well as the interaction between them are important factors in disease severity. In a paper by Wang et al [19], researchers found that area-level SDOH factors had an impact on mortality, including lower-income groups and areas with a higher proportion of essential workers, higher proportion of racially minoritized groups, and a higher proportion of apartment buildings. Another study based in South Korea found that lower SDOH status was associated with higher risk of infection [42]. In this study, Oh et al [42] found the lowest SDOH quartile to be associated with approximately a 20% increase in risk of infection compared to the highest quartile. Although these studies identify how SDOH disparities affect the risk of being infected with COVID-19 and subsequent severity outcomes, they do not further stratify by VOC—something our study was able to assess. Of particular interest in our cohort were differences between populations infected with Alpha and Gamma as these variants cocirculated in BC. During this period, there was a positive relationship between the proportion of essential workers and Gamma infections but an opposite trend for Alpha infections (Table 3), indicating that, during the same period, different VOCs were circulating more frequently among high and low proportions of racially minoritized groups, leading to the differential proportion of infections among hospitalized individuals.

This investigation has several key strengths. We were able to access the BCC19C, allowing for the analysis of a large population-based unbiased sample of >90,000 sequenced COVID-19 infections characterized mostly through WGS throughout the 2021 calendar year in the province of BC, where the COVID-19 pandemic’s main VOCs were present. We were able to compare robust estimates of morbidity and mortality following primary infection across several SDOH factors and vaccination levels along with linked comorbidity. This let us have minimal misclassification bias as well as adjust for key confounding variables on the pathway between VOC and severity. Many studies have had to rely on comparing waves in which certain VOCs were dominant or on VOC screening, both of which are subject to large misclassification bias. Linkage to SDOH variables also allowed us to adjust for key risk factors, including socioeconomic status (household income), structural racism (via proportion of minoritized groups), occupation (via proportion of essential workers), and sources of transmission (via housing density), which we have seen to be associated with both being infected with certain VOCs and with severity outcomes in our study and other Canadian investigations [43].

Selection bias may have been introduced from the sequencing strategy; however, this bias is strongly alleviated given the provincial laboratory’s goal of sequencing an unbiased representative sample of all individuals diagnosed using the polymerase chain reaction test. Furthermore, to be included in our study, individuals needed to have undergone COVID-19 testing, which may represent another selection bias to those who have access the medical system, although the province provides universal health coverage to all its residents. Our study relied on the most up-to-date census data to derive neighborhood-level SDOH measures. Despite not using patient-level data for SDOHs, the granular area-level data may be a better way to reflect community-level risk of infection and subsequent severity outcomes. Finally, there remains the possibility of capturing incidental hospitalization. We attempted to account for this by changing the definition of the hospitalization window and found there to only be a notable change when excluding those hospitalized on the same day as the laboratory collection date; however, this change did exclude 40.92% (2190/5352) of hospitalizations, including a differential exclusion among VOCs, and may have introduced other biases. Our previous analysis confirmed this unbiased sampling during December 2021, when the sequencing strategy changed in response to the sudden rise in Omicron infections [10].

After adjustment for demographic variables, SDOHs, vaccination status, and underlying comorbidities, we report Delta to be associated with the most severe outcomes. The proportion of hospitalized individuals was higher in those unvaccinated than in those fully vaccinated, further highlighting the importance of continued vaccination. We further reported that SDOHs had a differential distribution by VOC, which begs the need to further tease out the role of these factors on the association between VOC infection and severe outcomes.

However, it is important to consider the other sources of transmission dynamics beyond how SDOHs can operate in the pathway between viral infection and severe outcomes. For instance, in Canada, Oloye et al [44] demonstrated the role of wastewater as a sentinel surveillance tool in smaller communities following initial detection of the Delta VOC in a larger city within the same province, which showcases the potential limitation of relying solely on individuals who are tested throughout the pandemic who typically reside in large urban centers with readily available testing sites. Nevertheless, this study lays the groundwork for analyses aimed at teasing the contribution and role of VOCs in the exacerbated morbidity and mortality among socioeconomically at-risk populations. It also aligns with the call to not only consider vaccination for pandemic preparedness but to consider the socioeconomic effects, diversity, and infectivity of new variants as viruses evolve, as well as environmental effects [36]. Our study did not control directly for the effect of the environment, specifically air pollution, on infectivity and morbidity of the virus, which were demonstrated in an Italian study [3]; however, the inclusion of the 5 regional health authorities may serve as a crude proxy for environmental factors as the climate and topography of the Lower Mainland (Fraser and coastal areas) are distinct from those found in the north and in the interior of the province. Pandemic preparedness is not limited to active surveillance of cases and implementation of public health measures and vaccination, it also requires environmental monitoring of animal reservoirs. Indeed, in Ontario, Canada, Pickering et al [45] were able to show evidence not only of sustained evolution in wild animals but also of animal-to-human transmission.

### Conclusions

In conclusion, this study highlights the importance of targeted testing and sequencing to ensure timely detection and accurate estimation of severity in emerging VOCs, as well as the continued support for vaccination programs to lower the burden on health care systems and increase public health resilience in the context of infectious disease pandemics. Prioritized allocation of resources and services for areas of lower socioeconomic status and with a higher proportion of minoritized groups and highly dense housing should be tailored as these represent local drivers of inequalities in transmission in response to the resurgence of SARS-CoV-2.

## Appendix

Multimedia Appendix 1 Data sets available in British Columbia COVID-19 cohort, screening and sequencing strategy over time, definitions for covariates included in the analysis, study population demographics by covariates included in the analysis, and mortality model results.

## Abbreviations

aHR: adjusted hazard ratio
BC: British Columbia
BCC19C: British Columbia COVID-19 Cohort
BCCDC: British Columbia Centre for Disease Control
ICU: intensive care unit
IPTW: inverse probability treatment weight
qPCR: quantitative polymerase chain reaction
SDOH: social determinant of health
VOC: variant of concern
WGS: whole genome sequencing

## Data Availability

The data sets analyzed during this study through the British Columbia Covid-19 Cohort are available from the corresponding author on reasonable request. The genomic sequencing data sets generated during this study are available in the Global Initiative on Sharing All Influenza Data repository under the submitter BC Centre for Disease Control Public Health Laboratory [46]. The code used for the study models and whole genome sequencing analysis pipelines will be made available upon request to the corresponding author.
