# Supplementary material for: The Clinical Severity of COVID-19 Variants of Concern: Retrospective Population-Based Analysis
Source: JMIR Public Health Surveill. 2024 Aug 27;10:e45513. doi: 10.2196/45513 (PMC11387920; doi:10.2196/45513)
Supplement: Multimedia Appendix 1 [file publichealth_v10i1e45513_app1.docx]

Supplementary material

**BCC19C data information and variable definition**

Table S1: Datasets integrated within the BC COVID-19 Cohort

| Datasets | Data Date Ranges |
| --- | --- |
| **British Columbia Centre for Disease Control (BCCDC), Provincial Heath Services** | |
| **Authority (PHSA) and Regional Health Authority data sources:** | |
| Integrated COVID-19 laboratory dataset (SARS-CoV2 tests from private/public labs, includes sequencing and screening data) S1 | Jan 2020-onward |
| COVID-19 surveillance case data (information collected on all probable/confirmed cases as part of public health follow up) S2 | Jan 2020-onward |
| Provincial COVID-19 Monitoring Solution (critical and non-critical care hospital census data) S3 | Jan 2020-onward |
| Provincial Immunizations Registry (COVID-19 vaccination data) S4 | Dec 2020-onward |
| Provincial Laboratory Information Solution (laboratory tests from private/public labs) S5 | Jan 2020-onward |
| Public Health Reporting Data warehouse (Influenza laboratory tests) S6 | Jan 2008-onward |
| **Ministry of Health (MoH) Administrative Data Sources:** | |
| Emergency department visits (hospital-based and community-based ambulatory care) | Mar 2020-onward |
| Ministry of Health (MoH) Administrative Data Sources: | Data Date Ranges: |
| Client Roster (CR) (registry of enrollment in the universal public health insurance plan including residential history) S7 | 2008/9-onward |
| Discharge Abstracts Database (DAD) (hospital discharge records) S8 | 2008/9-onward |
| Medical Services Plan (MSP) (physician diagnostic and billing data for services provided through universal public health insurance plan) S9 | 2008/9-onward |
| PharmaNet (Pharma) (prescription drugs dispensed from community pharmacies, includes medications covered by public and private insurance plans) S10 | 2008/9-onward |
| BC Vital Statistics (VS) (deaths registry) S11 | 2008/9-onward |
| National Ambulatory Care Reporting System (NACRS) (hospital-based and community-based ambulatory care) S12 | 2011/12–onward |
| Chronic Disease Registry S13 | 2008/9-2018/19 |
| 811 Calls (respiratory calls only) S14 | 2014-onward |
| Health System Matrix S15 | 2018/19-onward |
| Population Grouper Methodology S16 | 2008/9-onward |

**References (Data Sources for eTable 1 above)**
S1. British Columbia Centre for Disease Control [creator]. Integrated COVID-19 laboratory dataset (SARS-CoV2 tests from private/public labs). Public Health Reporting Data Warehouse, British Columbia Centre for Disease Control [publisher] (2020). 2021.
S2. British Columbia Centre for Disease Control [creator]. COVID-19 surveillance case data. British Columbia Centre for Disease Control [publisher]. (2020). 2021.
S3. Provincial Health Services Authority [creator]. Provincial COVID-19 Monitoring Solution. Provincial Health Services Authority [publisher]. (2020). 2021.
S4. Provincial Health Services Authority [creator]. Provincial Public Health Information Systems [publisher]. (2020). 2021.
S5. Provincial Health Services Authority [creator]. COVID-19 vaccination data. Provincial Immunizations Registry, Provincial Public Health Information Systems [publisher]. (2020). 2021.
S6. British Columbia Centre for Disease Control [creator]. Respiratory datamart, Public Health Reporting Data Warehouse, British Columbia Centre for Disease Control [publisher] (2020). 2021.
S7. British Columbia Ministry of Health [creator]. Client Roster (Client Registry System/Enterprise Master Patient Index). British Columbia Ministry of Health [publisher]. Data Extract. MOH (2020). 2021. <https://www2.gov.bc.ca/gov/content/health/health-forms/online-services>
S8. British Columbia Ministry of Health [creator]. Discharge Abstract Database (Hospital Separations). British Columbia Ministry of Health [publisher]. Data Extract. MOH (2020). 2021. <https://www2.gov.bc.ca/gov/content/health/health-forms/online-services>
S9. British Columbia Ministry of Health [creator]. Medical Services Plan (MSP) Payment Information File. British Columbia Ministry of Health [publisher]. Data Extract. MOH (2020). 2021.https://www2.gov.bc.ca/gov/content/health/health-forms/online-services
S10. British Columbia Ministry of Health [creator]. PharmaNet. British Columbia Ministry of Health [publisher]. Data Extract. MOH (2020). 2021. <https://www2.gov.bc.ca/gov/content/health/health-forms/online-services>
S11. BC Vital Statistics Agency [creator]. Vital Statistics Deaths. BC Vital Statistics Agency [publisher]. Data Extract. BC Vital Statistics Agency (2020). 2021. <https://www2.gov.bc.ca/gov/content/health/health-forms/online-services>
S12. British Columbia Ministry of Health [creator]. National Ambulatory Care Reporting System. British Columbia Ministry of Health [publisher]. Data Extract. MOH (2020). 2021. <https://www2.gov.bc.ca/gov/content/health/health-forms/online-services>
S13. British Columbia Ministry of Health [creator]. Chronic Disease Registry. British Columbia Ministry of Health [publisher]. Data Extract. MOH (2020). 2020.
<https://www2.gov.bc.ca/gov/content/health/health-forms/online-services>
S14. British Columbia Ministry of Health [creator]. 811 calls. British Columbia Ministry of Health [publisher]. Data Extract. MOH (2020). 2021.
<https://www2.gov.bc.ca/gov/content/health/health-forms/online-services>
S15. British Columbia Ministry of Health [creator]. Health System Matrix. British Columbia Ministry of Health [publisher]. Data Extract. MOH (2020). 2021.
<https://www2.gov.bc.ca/gov/content/health/health-forms/online-services>
S16. British Columbia Ministry of Health [creator]. Population Grouper Methodology. British Columbia Ministry of Health [publisher]. Data Extract. MOH (2020). 2021.
<https://www2.gov.bc.ca/gov/content/health/health-forms/online-services>

Table S2. Screening and sequencing strategy over time

| Time period | Associated Pangolin lineages |
| --- | --- |
| Jan – May 2021 | A subset of samples was tested by targeted single nucleotide polymorphism (SNP) qPCR for VOC screening, followed by confirmation by WGS |
| Jun – Sep 2021 | Sample VOC status was determined by WGS alone |
| Sep – Nov 14 2021 | Owing to increased case burden and limited capacity, there was a transition to monthly period-limited prevalence (100% of positive samples on the first week of each month) and for the rest of the month a subset of targeted surveillance samples (outbreaks, vaccine escape, reinfection, and travel-related samples) in addition to a representative baseline surveillance of at least 10% of the general population with qPCR positive results. |
| Nov 15 – Dec 21 2021 | In the context of the Omicron variant emergence, WGS was resumed for all samples. |
| Dec 21 – Dec 31 2021 | A transition from full sequencing to sequencing a subset of representative positive samples in addition to priority cases (including outbreaks, long-term care, vaccine escape, travel-related, hospitalisation. |

Table S3: Definitions for covariates included in the analysis

| Variable | Data source | | Definition |
| --- | --- | --- | --- |
| **General** | | | |
| Age | COVID-19 Case Surveillance data, Client Roster | | Age in years at time of laboratory collection date. For modeling this variable was categorized as 0-4, 5-11, 11-19, 20-29, 30-39, 40-49, 50-59, 60-69, 70-79, and 80+. Data taken from COVID-19 case surveillance data and if missing, supplemented with Client Roster data. |
| Sex | COVID-19 Case Surveillance data, Client Roster | | Sex (male/female). Data taken from COVID-19 case surveillance data and if missing, supplemented with Client Roster data. |
| **Geography** | | | |
| Health authority | COVID-19 Case Surveillance data, Client Roster | | BC’s 5 Health Authorities: Fraser, Vancouver Coastal, Vancouver Island, Interior, Northern. Based on health authority of residence from COVID-19 Case Surveillance data and if missing, it was supplemented from the client roster. |
| **Vaccination status** | | | |
| Vaccination status | Provincial Immunization Registry | | This is defined as fully vaccinated, partially vaccinated, or unvaccinated. To be qualified as fully vaccinated or partially vaccinated, the vaccine must have been received =14 days prior to the lab collection date. If the vaccine was received within 14 days of diagnosis, this dose was not counted towards the vaccination status. To be considered fully vaccinated, 2 doses of Pfizer, AstraZeneca or Moderna (or any combination) needed to be received =14 days prior to laboratory collection date. If only 1 was received, individuals were considered partially vaccinated. Note, those who received Johnson and Johnson qualified as fully vaccinated after receiving only 1 dose. Those who received vaccines not provided in Canada, needed 2 doses to qualify as fully vaccinated. |
| Booster dose | Provincial Immunization Registry | | Booster doses were defined in a similar fashion to vaccination status. If a third (or second dose if Johnson and Johnson was original dose) dose of the any vaccine was received =14 days prior to laboratory collection date. This variable was coded as a binary variable, either having or not having received a booster dose. This variable was used for the sensitivity analysis performed in Table S6. |
| Time since full dose | Provincial Immunization Registry | | The amount of time passed since being fully vaccinated (as per definition above). For modeling, this was used a categorical variable with the following levels: <3 months, 2-6 months, >6 months or not fully vaccinated. This variable was used for the sensitivity analysis performed in Table S6. |
| **SDOH** | | | |
| Neighbourhood income quintile | Statistics Canada 2016 Census | | [Neighbourhood income quintile by dissemination area (DA) level. Quintile 5 is the wealthiest. Income quintiles were based on the average income per single-person home equivalent. The data was from the 2016 census. See link for more information on how neighbourhood income quintiles are calculated (https://www150.statcan.gc.ca/n1/pub/82-221-x/2013001/quality-qualite/qua8-eng.htm).](https://www150.statcan.gc.ca/n1/pub/82-221-x/2013001/quality-qualite/qua8-eng.htm) |
| Proportion of essential worker quintile | Statistics Canada 2016 Census | | Proportion of essential worker quintile by dissemination area (DA) level. Quintile 5 is the highest proportion of visible minority. This variable was calculated by dividing those working in the essential sector/industry by the total labour force. Essential industries include the following: trades, transport and equipment operators and related occupations; natural resources, agriculture and related production occupations; occupations in manufacturing and utilities; and sales and service occupations. The data was from the 2016 census. |
| Proportion of high-density housing quintile | Statistics Canada 2016 Census | | Proportion of high-density housing quintile by dissemination area (DA) level. Quintile 5 is the highest proportion of high density housing This variable was calculated by dividing the number of high density dwellings by the total number of dwellings in a given DA. High density housing referred to the following: Apartment in a building that has five or more storeys, other attached dwelling, row house, apartment or flat in a duplex, apartment in a building that has fewer than five storeys, apartment in a building what has fewer than five storeys, other single-attached house, and movable dwelling The data was from the 2016 census. |
| Proportion of visible minority quintile | Statistics Canada 2016 Census | | Proportion of visible minority quintile by dissemination area (DA) level. Quintile 5 is the highest proportion of visible minority. This variable was calculated by dividing the total number of people who identified as a visible minority by the total number of people living in the DA. Visible minorities include 13 derived groups: Aboriginal, Black, Filipino, Latin American, South Asian, Chinese, Arab, Southeast Asian, West Asian, Korean, Japanese, other visible minorities and multiple visible minorities. The data was from the 2016 census. See link for more information on: <https://www12.statcan.gc.ca/census-recensement/2016/ref/guides/006/98-500-x2016006-eng.cfm> |
| **Comorbidities** | | | |
| Elixhauser Index | DAD/MSP | | The Elixhauser Comorbidity Index is a method of categorizing co-morbidities of patients using ICD diagnosis codes captured in administrative data). In our study we captured these ICD codes from hospital discharge data (DAD) and physician billing data (MSP). The index is comprised of 30 co-morbidities, which are either coded as present or absent. The score indicates number of co-morbidities. For modeling, this variable was categorized as 0, 1, 2 and 3+. For more information on the Elixhauser Comorbidity Index, see: http://mchp-appserv.cpe.umanitoba.ca/viewConcept.php?printer=Y&conceptID=1436 and https://pubmed.ncbi.nlm.nih.gov/9431328/ |
| **Exposure** | | | |
| Variant of concern (via WGS) | Integrated COVID-19 Laboratory Dataset | | [Delta: B.1.617.2, AY.; Omicron: B.1.1.529, BA. As determined by WGS. The British Columbia Centre for Disease Control (BCCDC) Public Health Laboratory (PHL) uses a modified ARTIC Network bioinformatics protocol: https://github.com/BCCDC-PHL/ncov2019-artic-nf) to process reads, align to the SARS-CoV-2 reference genome and generate both variant calls and a consensus genome sequence.](https://github.com/BCCDC-PHL/ncov2019-artic-nf) |
| **Outcomes** | | | |
| Hospitlaisation | PCMS, COVID-19 Case surveillance data | COVID-19 hospitalization admission date within -2 to +14 days (inclusive) from COVID-19 lab collection date. Anything outside this range was not counted as an outcome. Two datasets were used to ascertain hospitalization status: case surveillance data (line-list of all COVID-19 diagnoses) and the PCMS (a daily census of hospitalizations). Two data sources were combined: 1) COVID-19 case surveillance data which includes an ever hospitalised flag and a hospital admission date, and 2) the Provincial COVID-19 Monitoring Solution (PCMS) which is a daily census of occupancy submitted by hospitals in BC and includes a field identifying whether a patient is COVID-19 positive Record of hospitalization in either dataset was sufficient. A hospitalization from the case surveillance dataset was defined as follows: “any person admitted to a hospital for at least an overnight stay, for reasons directly or indirectly related to their COVID-19 infection, and with no period of complete recovery between illness and admission. If unable to determine whether an admission was related to COVID-19, report as a hospital admission.” To derive relevant variables from the PCMS data (e.g., admission and discharge dates, length of stay, ICU admission), we rolled up the daily census records into episodes of care. An episode started with the first census record and ended with a discharge record or, if no discharge record was available, the last available census record. In the event of the latter, the last census record was used as a proxy for discharge date. Hospital transfers that occurred within 1 day of a discharge date were included within the same episode of care. | |
| ICU admissions | PCMS, COVID-19 Case surveillance data | Both hospital data sources were used to identify ICU admissions. The case surveillance data includes a field for ICU admission and the PCMS data includes a field for unit of care (e.g., ICU). ICU criteria was met if an individual was hospitalized within the –2 to 14 day time window and had record of an ICU admission associated with that hospitalization within the same dataset. ICU admission date was available in PCMS data but not the case surveillance data. | |
| Death | COVID-19 Case surveillance data | A death (from any cause) occurring in any person with no period of complete recovery between illness and death. Deaths within 30 days of lab collection date were considered to have met the criteria for COVID-19 death. | |

Table S4: Study population demographics

|  | **Alpha** | **Gamma** | **Delta** | **Omicron** | **Overall** |
| --- | --- | --- | --- | --- | --- |
|  | **(N=20,487)** | **(N=15,223)** | **(N=49,161)** | **(N=7,093)** | **(N=91,964)** |
| **Sex** |  |  |  |  |  |
| Female | 10,075 (49.2%) | 7,208 (47.3%) | 24,386 (49.6%) | 3,606 (50.8%) | 45,275 (49.2%) |
| Male | 10,412 (50.8%) | 8,015 (52.7%) | 24,775 (50.4%) | 3,487 (49.2%) | 46,689 (50.8%) |
| **Age (years)** |  |  |  |  |  |
| Mean (SD) | 36.6 (18.7) | 36.1 (18.0) | 35.7 (19.6) | 35.5 (16.0) | 35.9 (18.9) |
| Median [Min, Max] | 35.0 [3.00, 107] | 34.0 [3.00, 102] | 34.0 [3.00, 104] | 33.0 [3.00, 100] | 34.0 [3.00, 107] |
| **Age categories (years)** |  |  |  |  |  |
| 0-4 | 309 (1.5%) | 185 (1.2%) | 779 (1.6%) | 50 (0.7%) | 1,323 (1.4%) |
| 5-11 | 1,367 (6.7%) | 979 (6.4%) | 6,152 (12.5%) | 301 (4.2%) | 8,799 (9.6%) |
| 12-19 | 2,266 (11.1%) | 1,674 (11.0%) | 4,153 (8.4%) | 526 (7.4%) | 8,619 (9.4%) |
| 20-29 | 4,185 (20.4%) | 3,353 (22.0%) | 9,007 (18.3%) | 2,101 (29.6%) | 18,646 (20.3%) |
| 30-39 | 3,981 (19.4%) | 3,004 (19.7%) | 9,774 (19.9%) | 1,561 (22.0%) | 18,320 (19.9%) |
| 40-49 | 3,124 (15.2%) | 2,452 (16.1%) | 7,302 (14.9%) | 1,088 (15.3%) | 13,966 (15.2%) |
| 50-59 | 2,522 (12.3%) | 1,877 (12.3%) | 5,456 (11.1%) | 846 (11.9%) | 10,701 (11.6%) |
| 60-69 | 1,701 (8.3%) | 1,064 (7.0%) | 3,811 (7.8%) | 438 (6.2%) | 7,014 (7.6%) |
| 70-79 | 768 (3.7%) | 419 (2.8%) | 1,842 (3.7%) | 120 (1.7%) | 3,149 (3.4%) |
| 80+ | 264 (1.3%) | 216 (1.4%) | 885 (1.8%) | 62 (0.9%) | 1,427 (1.6%) |
| **Health Authority** |  |  |  |  |  |
| Fraser | 14,277 (69.7%) | 8,020 (52.7%) | 15,557 (31.6%) | 3,189 (45.0%) | 41,043 (44.6%) |
| Interior | 1,846 (9.0%) | 1,214 (8.0%) | 15,409 (31.3%) | 664 (9.4%) | 19,133 (20.8%) |
| Northern | 438 (2.1%) | 83 (0.5%) | 6,829 (13.9%) | 127 (1.8%) | 7,477 (8.1%) |
| Vancouver Coastal | 3,490 (17.0%) | 5,336 (35.1%) | 6,507 (13.2%) | 2,160 (30.5%) | 17,493 (19.0%) |
| Vancouver Island | 436 (2.1%) | 570 (3.7%) | 4,859 (9.9%) | 953 (13.4%) | 6,818 (7.4%) |
| **Vaccination status** |  |  |  |  |  |
| Not vaccinated | 18,809 (91.8%) | 13,612 (89.4%) | 30,448 (61.9%) | 526 (7.4%) | 63,395 (68.9%) |
| Partially vaccinated | 1,603 (7.8%) | 1,541 (10.1%) | 4,155 (8.5%) | 111 (1.6%) | 7,410 (8.1%) |
| Fully vaccinated | 75 (0.4%) | 70 (0.5%) | 14,558 (29.6%) | 6,456 (91.0%) | 21,159 (23.0%) |
| **Comorbidities** |  |  |  |  |  |
| 0 | 7,624 (37.2%) | 6,151 (40.4%) | 17,928 (36.5%) | 2,652 (37.4%) | 34,355 (37.4%) |
| 1 | 5,494 (26.8%) | 4,180 (27.5%) | 12,990 (26.4%) | 2,141 (30.2%) | 24,805 (27.0%) |
| 2 | 3,227 (15.8%) | 2,232 (14.7%) | 7,523 (15.3%) | 1,155 (16.3%) | 14,137 (15.4%) |
| 3+ | 4,142 (20.2%) | 2,660 (17.5%) | 10,720 (21.8%) | 1,145 (16.1%) | 18,667 (20.3%) |
| **Hospitalisation** |  |  |  |  |  |
| No hospital admission | 19,499 (95.2%) | 14,159 (93.0%) | 45,971 (93.5%) | 6,983 (98.4%) | 86,612 (94.2%) |
| Hospital admission | 988 (4.8%) | 1,064 (7.0%) | 3,190 (6.5%) | 110 (1.6%) | 5,352 (5.8%) |
| **ICU** |  |  |  |  |  |
| No ICU admission | 20,193 (98.6%) | 14,873 (97.7%) | 48,139 (97.9%) | 7,083 (99.9%) | 90,288 (98.2%) |
| ICU admission | 294 (1.4%) | 350 (2.3%) | 1,022 (2.1%) | 10 (0.1%) | 1,676 (1.8%) |
| **Death** |  |  |  |  |  |
| Alive | 20,416 (99.7%) | 15,161 (99.6%) | 48,734 (99.1%) | 7,088 (99.9%) | 91,399 (99.4%) |
| Died | 71 (0.3%) | 62 (0.4%) | 427 (0.9%) | <10 | 565 (0.6%) |
| **Income** |  |  |  |  |  |
| Q1 (Highest) | 3,081 (15.0%) | 2,958 (19.4%) | 9,164 (18.6%) | 1,761 (24.8%) | 16,964 (18.4%) |
| Q2 | 3,611 (17.6%) | 3,174 (20.9%) | 10,297 (20.9%) | 1,689 (23.8%) | 18,771 (20.4%) |
| Q3 | 4,394 (21.4%) | 3,094 (20.3%) | 9,630 (19.6%) | 1,423 (20.1%) | 18,541 (20.2%) |
| Q4 | 5,250 (25.6%) | 2,973 (19.5%) | 9,242 (18.8%) | 1,210 (17.1%) | 18,675 (20.3%) |
| Q5 | 4,151 (20.3%) | 3,024 (19.9%) | 10,828 (22.0%) | 1,010 (14.2%) | 19,013 (20.7%) |
| **Essential worker proportion** |  |  |  |  |  |
| Q1 (Lowest) | 2,724 (13.3%) | 3,225 (21.2%) | 6,061 (12.3%) | 2,071 (29.2%) | 14,081 (15.3%) |
| Q2 | 3,506 (17.1%) | 3,512 (23.1%) | 9,112 (18.5%) | 1,764 (24.9%) | 17,894 (19.5%) |
| Q3 | 3,884 (19.0%) | 3,008 (19.8%) | 10,729 (21.8%) | 1,437 (20.3%) | 19,058 (20.7%) |
| Q4 | 4,108 (20.1%) | 2,676 (17.6%) | 11,633 (23.7%) | 1,047 (14.8%) | 19,464 (21.2%) |
| Q5 | 6,265 (30.6%) | 2,802 (18.4%) | 11,626 (23.6%) | 774 (10.9%) | 21,467 (23.3%) |
| **Racially minoritised group proportion** |  |  |  |  |  |
| Q1 (Lowest) | 1,038 (5.1%) | 662 (4.3%) | 10,997 (22.4%) | 537 (7.6%) | 13,234 (14.4%) |
| Q2 | 1,886 (9.2%) | 1,792 (11.8%) | 12,967 (26.4%) | 878 (12.4%) | 17,523 (19.1%) |
| Q3 | 3,016 (14.7%) | 2,853 (18.7%) | 10,824 (22.0%) | 1,496 (21.1%) | 18,189 (19.8%) |
| Q4 | 4,947 (24.1%) | 4,412 (29.0%) | 7,995 (16.3%) | 2,278 (32.1%) | 19,632 (21.3%) |
| Q5 | 9,600 (46.9%) | 5,504 (36.2%) | 6,378 (13.0%) | 1,904 (26.8%) | 23,386 (25.4%) |
| **High-density housing proportion** |  |  |  |  |  |
| Q1 (Lowest) | 1,613 (7.9%) | 1,257 (8.3%) | 9,505 (19.3%) | 796 (11.2%) | 13,171 (14.3%) |
| Q2 | 2,590 (12.6%) | 2,090 (13.7%) | 10,430 (21.2%) | 1,121 (15.8%) | 16,231 (17.6%) |
| Q3 | 3,910 (19.1%) | 2,838 (18.6%) | 10,901 (22.2%) | 1,452 (20.5%) | 19,101 (20.8%) |
| Q4 | 6,020 (29.4%) | 3,867 (25.4%) | 9,400 (19.1%) | 1,637 (23.1%) | 20,924 (22.8%) |
| Q5 | 6,354 (31.0%) | 5,171 (34.0%) | 8,925 (18.2%) | 2,087 (29.4%) | 22,537 (24.5%) |

Table S5: Full IPTW Cox PH model for mortality

| Variable | Hospitalisation | ICU admission | Mortality |
| --- | --- | --- | --- |
| **Variant (ref = Alpha)** |  |  |  |
| Delta | 2 (1.92, 2.08) | 2.05 (1.91, 2.20) | 3.7 (3.23, 4.25) |
| Gamma | 1.43 (1.38, 1.49) | 1.8 (1.69, 1.93) | 1.33 (1.15, 1.54) |
| Omicron | 1.46 (1.36, 1.56) | 0.73 (0.61, 0.86) | 1.28 (1.01, 1.61) |
| **Age (ref = 20-29)** |  |  |  |
| 0-4 | 0.15 (0.09, 0.24) | 0.12 (0.04, 0.39) | 0 (0, NA) |
| 2-4 | 0.16 (0.13, 0.20) | 0.01 (0, 0.06) | 0 (0, NA) |
| 5-11 | 0.38 (0.33, 0.45) | 0.53 (0.4, 0.70) | 1.58 (0.57, 4.34) |
| 30-39 | 2.03 (1.88, 2.18) | 2.5 (2.16, 2.88) | 6.5 (3.32, 12.72) |
| 40-49 | 3.38 (3.15, 3.63) | 4.74 (4.14, 5.44) | 4.38 (2.17, 8.85) |
| 50-59 | 4.74 (4.42, 5.08) | 6.54 (5.71, 7.49) | 15.08 (7.83, 29.05) |
| 60-69 | 8.39 (7.83, 8.99) | 13.3 (11.63, 15.20) | 40.97 (21.5, 78.06) |
| 70-79 | 15.25 (14.18, 16.41) | 20.49 (17.81, 23.58) | 125.5 (66.07, 238.39) |
| 80+ | 35.14 (32.57, 37.90) | 18.13 (15.44, 21.30) | 444.77 (234.44, 843.77) |
| **Sex (ref = Female)** |  |  |  |
| Male | 1.34 (1.3, 1.38) | 1.59 (1.51, 1.68) | 1.83 (1.65, 2.02) |
| **Comorbidities (ref = 0)** |  |  |  |
| 1 | 1.15 (1.09, 1.21) | 1.04 (0.95, 1.14) | 1.33 (0.95, 1.86) |
| 2 | 1.26 (1.2, 1.33) | 0.91 (0.83, 1.00) | 2.46 (1.8, 3.35) |
| 3+ | 2.31 (2.21, 2.42) | 2.05 (1.9, 2.22) | 6.79 (5.15, 8.94) |
| **Geography (ref = Fraser)** |  |  |  |
| Interior | 0.99 (0.94, 1.04) | 0.92 (0.84, 1.01) | 1.27 (1.08, 1.48) |
| Northern | 1.24 (1.17, 1.31) | 2.26 (2.07, 2.48) | 0.88 (0.7, 1.11) |
| Vancouver Coastal | 1.05 (1, 1.10) | 0.96 (0.88, 1.04) | 1.29 (1.1, 1.52) |
| Vancouver Island | 0.84 (0.79, 0.91) | 0.94 (0.83, 1.06) | 1.15 (0.91, 1.44) |
| **Income (ref = Q1)** |  |  |  |
| Q2 | 0.49 (0.47, 0.52; p = <0.001) | 0.52 (0.48, 0.56; p = <0.001) | 0.67 (0.59, 0.76; p = <0.001) |
| Q3 | 0.92 (0.88, 0.97; p = <0.001) | 0.98 (0.89, 1.08; p = 0.7) | 1.16 (0.94, 1.43; p = 0.17) |
| Q4 | 0.99 (0.93, 1.04; p = 0.62) | 0.91 (0.82, 1.01; p = 0.08) | 1.03 (0.83, 1.29; p = 0.77) |
| Q5 | 0.99 (0.93, 1.05; p = 0.71) | 1.27 (1.15, 1.41; p = <0.001) | 1.24 (0.99, 1.55; p = 0.06) |
| **Essential worker (ref = Q1)** |  |  |  |
| Q2 | 1.29 (1.21, 1.37; p = <0.001) | 1.54 (1.39, 1.72; p = <0.001) | 1.38 (1.1, 1.74; p = 0.01) |
| Q3 | 1.13 (1.07, 1.19; p = <0.001) | 0.99 (0.89, 1.09; p = 0.83) | 1.03 (0.83, 1.27; p = 0.8) |
| Q4 | 1.09 (1.03, 1.16; p = <0.001) | 1.13 (1.02, 1.26; p = 0.02) | 1.59 (1.3, 1.96; p = <0.001) |
| Q5 | 1.25 (1.18, 1.33; p = <0.001) | 1.24 (1.12, 1.38; p = <0.001) | 1.23 (0.99, 1.53; p = 0.06) |
| **Visible minority (ref = Q1)** |  |  |  |
| Q2 | 1.14 (1.08, 1.21; p = <0.001) | 1.18 (1.06, 1.31; p = <0.001) | 1.72 (1.39, 2.14; p = <0.001) |
| Q3 | 0.98 (0.93, 1.03; p = 0.36) | 1.22 (1.12, 1.32; p = <0.001) | 1.04 (0.88, 1.23; p = 0.62) |
| Q4 | 0.98 (0.93, 1.04; p = 0.54) | 1.02 (0.93, 1.12; p = 0.66) | 1.23 (1.03, 1.47; p = 0.02) |
| Q5 | 1.14 (1.07, 1.22; p = <0.001) | 1.18 (1.06, 1.32; p = <0.001) | 1.05 (0.85, 1.31; p = 0.65) |
| **Housing density (ref = Q1)** |  |  |  |
| Q2 | 1.12 (1.05, 1.20; p = <0.001) | 1.13 (1.01, 1.26; p = 0.04) | 0.86 (0.7, 1.08; p = 0.19) |
| Q3 | 0.9 (0.85, 0.95; p = <0.001) | 0.93 (0.84, 1.02; p = 0.1) | 1.3 (1.06, 1.59; p = 0.01) |
| Q4 | 1.08 (1.02, 1.13; p = 0.01) | 1 (0.91, 1.10; p = 0.99) | 1.24 (1.01, 1.52; p = 0.04) |
| Q5 | 1.05 (0.99, 1.12; p = 0.08) | 0.89 (0.81, 0.99; p = 0.03) | 1.33 (1.07, 1.65; p = 0.01) |
| **Vaccination (ref = Unvaccinated)** |  |  |  |
| Partially vaccinated | 1.08 (1.01, 1.15; p = 0.02) | 0.99 (0.89, 1.11; p = 0.87) | 1.46 (1.16, 1.84; p = <0.001) |
| Vaccinated | 0.2 (0.19, 0.21; p = <0.001) | 0.13 (0.11, 0.15; p = <0.001) | 0.15 (0.13, 0.18; p = <0.001) |

Table S6: Sensitivity analysis for Cox regression models given hospitalisation definition

|  |  | **-2-14 days** |  | **0-14 days** |  | **1-14 days** |
| --- | --- | --- | --- | --- | --- | --- |
| **Variant** | **n** | **aHR (95% CI)** | **n** | **aHR (95% CI)** | **n** | **aHR (95% CI)** |
| **Full cohort** | **5,323** |  | **5,195** |  | **3,585** |  |
| Alpha | 988 |  | 962 |  | 709 |  |
| Gamma | 1,064 | 1.66 (1.52 - 1.82) | 1,051 | 1.69 (1.54 - 1.85) | 849 | 1.93 (1.74 - 2.13) |
| Delta | 3,190 | 2.18 (2.01 - 2.36) | 3,078 | 2.18 (2.01 - 2.37) | 1,971 | 1.76 (1.59 - 1.94) |
| Omicron | 110 | 1.55 (1.25 - 1.92) | 104 | 1.55 (1.24 - 1.92) | 56 | 1.15 (0.86 - 1.54) |
| **Unvaccinated** | **4,237** |  | **4,132** |  | ***Masked*** |  |
| Alpha | 866 |  | 846 |  | 646 |  |
| Gamma | 915 | 1.71 (1.55 – 1.88) | 908 | 1.73 (1.57 - 1.9) | 760 | 1.96 (1.76 - 2.18) |
| Delta | 2,441 | 2.29 (2.09 - 2.5,) | 2,365 | 2.27 (2.08 - 2.49) | 1,552 | 1.85 (1.67 - 2.06) |
| Omicron | 15 | 1.78 (1.06 - 2.96) | 13 | 1.59 (0.92 - 2.76) | <10 | Masked |

*n = number of hospitalisations meeting the given outcome definition* *All hospitalisations that did not meet this definition were excluded* **Models not weighted using IPTW"***.** Models are masked for severity outcomes under 10.
